# Mental health consequences of the 2024 Feni flash flood in Bangladesh: prevalence and risk factors

**DOI:** 10.3389/fpubh.2025.1687943

**Published:** 2025-12-08

**Authors:** Md Mostafizur Rahman, Samantha Alam, Ifta Alam Shobuj, Md. Mehedi Hasan Santo, Md. Tanvir Hossain, Farzana Rahman, Md. Kaium Hossain, Edris Alam, Md. Kamrul Islam

**Affiliations:** 1 Department of Disaster Management and Resilience, Faculty of Arts and Social Sciences, Bangladesh University of Professionals, Dhaka, Bangladesh; 2 Sociology Discipline, Social Science School, Khulna University, Khulna, Bangladesh; 3 Department of Computer Science and Engineering, Independent University, Dhaka, Bangladesh; 4 School of Business and Economics, United International University, Dhaka, Bangladesh; 5 Department of Geography and Environmental Studies, University of Chittagong, Chittagong, Bangladesh; 6 Faculty of Resilience, Rabdan Academy, Abu Dhabi, United Arab Emirates; 7 Department of Civil and Environmental Engineering, College of Engineering, King Faisal University, AlAhsa, Saudi Arabia

**Keywords:** mental health, flash flood, psychological distress, disaster resilience, Bangladesh, disaster preparedness

## Abstract

The 2024 flash flood in Feni District, Bangladesh, was an unforeseen disaster that significantly disrupted livelihoods, infrastructure, and overall well-being. While the physical and economic consequences of floods are widely studied, their impact on mental health remains underexplored, particularly in flood-prone regions like Bangladesh. This study examines the mental health issue experienced by flood survivors, focusing on depression, anxiety, and stress using the Depression, Anxiety, and Stress Scale-21 (DASS-21). A cross-sectional survey was conducted among 1,981 individuals affected by the flood. The findings reveal an alarming prevalence of mental health disorders, with 52.80% suffering from severe depression, 44.17% from severe anxiety, and 46.90% from severe stress. Key risk factors included age, education level, chronic illness, and social satisfaction. Older adults and individuals with lower educational attainment or pre-existing health conditions were particularly vulnerable to mental health challenges. The study also identified gaps in disaster preparedness, including ineffective early warning systems, inadequate evacuation shelters, and insufficient socioeconomic support. These findings underscore the pressing need for targeted mental health interventions, strengthened disaster management policies, and intensified community resilience efforts. Strengthening mental health services, improving flood preparedness, and fostering community support networks are critical to mitigating the long-term psychological impact of future disasters.

## Introduction

Floods are among the most frequent and devastating natural hazards worldwide, causing extensive damage to infrastructure, livelihoods, and human wellbeing (1, 2). In recent years, the increasing frequency and intensity of floods have been closely linked to climate change, unplanned urbanization, and environmental degradation (3). Bangladesh, due to its geographical location in the delta of major rivers, remains one of the most flood-prone countries, with a large population exposed to high flood risk (4–7).

The 2024 flash flood in Feni District, Bangladesh, was a catastrophic event that significantly disrupted daily life, leading to widespread displacement, economic losses, and infrastructural damage (8, 9). Unlike the annual monsoon floods that the country typically experiences, this event was sudden and unexpected, exacerbating the challenges faced by affected communities (10). The flood submerged vast areas, destroyed homes and agricultural lands, and severely affected local economies, leaving thousands of people in a state of prolonged distress (9). While immediate concerns following such disasters often focus on physical damage and economic losses, the mental health impacts remain critically underexplored in the Bangladeshi context.

Exposure to natural hazards is known to elevate the risk of mental health disorders, including post-traumatic stress disorder (PTSD), depression, anxiety, and emotional distress (11, 12). Vulnerable groups such as women, children, older adults, and individuals with pre-existing health conditions are particularly susceptible to the psychological consequences of flooding (4). Studies found that flood survivors in Bangladesh experienced significant mental health challenges, with symptoms persisting long after the disaster (4, 13). Furthermore, poor access to mental health care and inadequate disaster preparedness exacerbate these challenges, leaving many affected individuals without the necessary psychological support (14).

The impact of floods on mental health is not limited to immediate trauma but extends to long-term socioeconomic stressors, such as loss of livelihoods, displacement, and social isolation. Research and report in Australia following severe flooding events found a significant increase in mental health among affected individuals, highlighting the need for long-term mental health interventions (15–17). Similar patterns have been observed in South Asia, where disaster-related mental health issues remain largely unaddressed due to resource constraints and social stigma surrounding mental health care (18).

This study aims to bridge the knowledge gap regarding the mental health impacts of floods in Bangladesh by assessing the depression, anxiety, and stress experienced by residents of Feni District following the 2024 flash flood. Using validated tools such as the Depression, Anxiety, and Stress Scale-21 (DASS-21) (19). This research investigates the prevalence and severity of mental health conditions among flood-affected individuals. Additionally, it examines key sociodemographic and environmental factors influencing mental health outcomes, including age, education level, social support, and prior flood experience.

By adopting a quantitative approach with a large sample size, this study provides valuable insights into the mental health consequences of flooding in Bangladesh. The findings are expected to inform disaster response strategies, emphasizing the need for targeted mental health interventions and enhanced preparedness measures to mitigate the psychological impact of future floods. Strengthening social networks, improving disaster education, and ensuring accessible mental health services are crucial steps toward building resilience in flood-prone communities.

## Methods

### Research design

This study employs a cross-sectional design to assess the mental health impact of the 2024 flash flood on adult residents (18 years or older) of three flood-affected Upazilas (Sonagazi, Chhagalnaiya, and Fulgazi) in Feni District, Bangladesh. In Bangladesh, an upazila refers to an administrative sub-district, typically consisting of several unions and villages. The DASS-21 were employed as the tool for assessing mental health problems (19). The unprecedented scale of the flood prompted the hypothesis that significant mental health issues, including depression, anxiety, and stress, may have arisen among the affected population.

### Study area

Feni, which was previously a subdivision of Noakhali District, was designated as a district on March 1, 1984 (20). It is situated between 22°44′ and 23°17′ north latitudes and 91°15′ and 91°35′ east longitudes. The district shares its northern border with Comilla District and India, its eastern boundary with India and Chattogram District, its southern border with Chattogram and Noakhali Districts, and its western boundary with Noakhali District.

Fulgazi Upazila is located in the northern part of Feni District, covering an area of approximately 102.19 km^2^ (21). It is bordered by Tripura, India, to the east and is characterized by its fertile floodplains, which are traversed by rivers such as the Muhuri et al. (22). The population of Fulgazi is around 119,558, with a literacy rate of approximately 60% (22). Historically, Fulgazi has been prone to flooding due to its low-lying geography and proximity to major rivers. For instance, the 1998 floods caused significant damage to settlements and agricultural areas in the region (23). The recent floods in 2024 have further highlighted this vulnerability, with reports indicating that over 40 villages were inundated, affecting thousands of residents (24, 25).

Chhagalnaiya Upazila spans about 139.59 km^2^ and has a population of approximately 187,156 (22). It is situated adjacent to Fulgazi and features a similar topography, making it susceptible to flooding. In August 2024, Chhagalnaiya experienced severe flooding that submerged numerous villages, leaving many residents stranded. The water levels in local rivers surpassed danger marks due to heavy rainfall and upstream runoff from India (24, 25). This upazila’s vulnerability is compounded by its reliance on agriculture, as floods can devastate crops and disrupt livelihoods.

**Figure 1:**
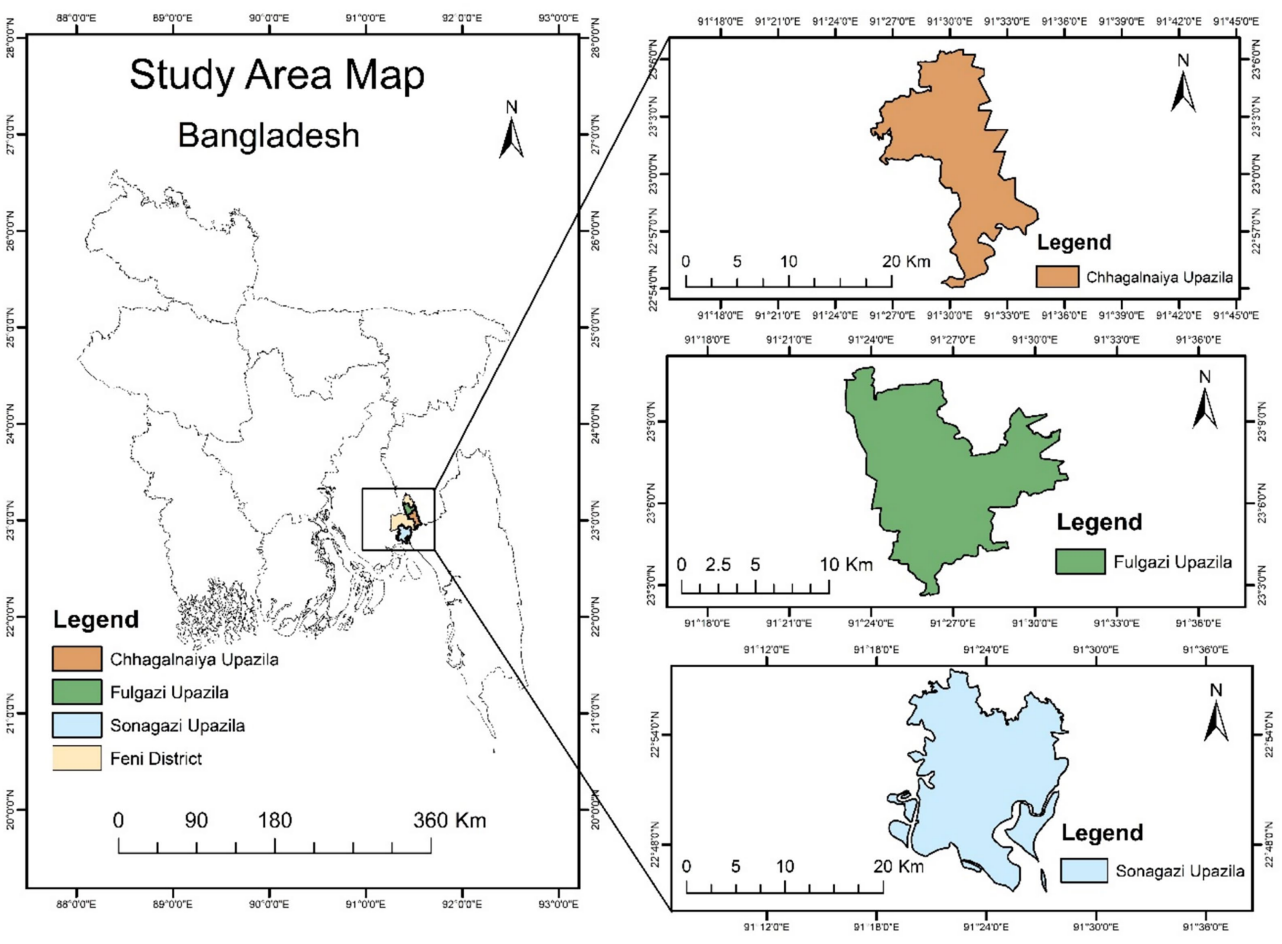
Study area (27).

Sonagazi Upazila covers an area of about 284.89 km^2^ and has a population of approximately 262,547 (22). It shares borders with both Fulgazi and Chhagalnaiya, making it part of a continuous flood-prone region. Sonagazi has also faced significant flooding challenges in recent years. In August 2024, it was reported that approximately 350,000 people across Sonagazi, Fulgazi, and Chhagalnaiya were affected by rising waters, resulting in severe disruptions to daily life and infrastructure (24–26). Flooding in this region often results in road submersion and loss of access to essential services (see Figure 1).

### Survey techniques

The survey was conducted in Bengali, the local language, and utilized the DASS-21 (19) to assess the mental health impact on individuals affected by the 2024 flood in the study areas. We used a validated Bengali version of the DASS-21 (28), which we had previously applied in studies during the COVID-19 pandemic (29) and the 2022 flood (4). The DASS-21, a condensed version of the original DASS-42, comprises 21 items divided into three subscales, each containing seven items measuring depression, anxiety, and stress. This tool has been widely used in various studies (29–31) to assess emotional distress in adults. Each item in the DASS-21 is rated on a four-point Likert scale: 0 (“Did not apply to me at all”), 1 (“Applied to me to some degree or some of the time”), 2 (“Applied to me to a considerable degree or a good part of the time”), and 3 (“Applied to me very much or most of the time”). Participants reported their symptoms based on their experiences over the past week. Scores for depression, anxiety, and stress were computed by summing the respective item scores and multiplying them accordingly (depression: items 3, 5, 10, 13, 16, 17, 21; stress: items 1, 6, 8, 11, 12, 14; anxiety: items 2, 4, 7, 9, 15, 19, 20). The DASS-21 categorizes severity into five levels: normal, mild, moderate, severe, and extremely severe (Table 1). It serves as an effective tool for assessing the severity of emotional distress and potential treatment responses.

**Table 1:** Cut-off values for the DASS-21’s labels for depression, anxiety, and stress (19).

Severity Label	Depression	Anxiety	Stress
Normal	0–9	0–7	0–14
Mild	10–13	8–9	15–18
Moderate	14–20	10–14	19–25
Severe	21–27	15–19	26–33
Extremely severe	28+	20+	34+

A subset of the study participants participated in a pilot survey, and their feedback was used to refine the final version of the questionnaire. However, data from this initial survey were excluded from the final analysis. To evaluate the reliability of the three pretested DASS sections, Cronbach’s alpha was calculated, with all sections achieving values above 0.75. Generally, a Cronbach’s alpha above 0.60 indicates good internal consistency (32, 33).

The questionnaire was divided into four main sections. The first section covered key sociodemographic factors, including gender, age group, marital status, education level, location, housing type, presence of vulnerable family members, and any chronic illnesses. We also asked participants about their social satisfaction with the question, “How do you perceive your current social life?” The second section focused on flood-related inquiries, while the third section explored flood-related damages; the final section consisted of the DASS-21.

Sociodemographic data and flood-related loss and damage were considered as independent variables, hypothesized to influence all three DASS aspects. Although the questionnaire was originally designed as a self-reported tool (19), most participants were either illiterate or had low levels of education. To collect self-reported data, we conducted face-to-face interviews, asking questions in a clear and understandable manner. Previous studies have also employed the DASS-21 in face-to-face interviews (4, 34–36). Drawing on our prior experience working with participants in remote areas, we ensured that the questions were simple and easy to comprehend, and we made improvements to the questionnaire format based on our pilot survey.

### Data management

In February 2025, a survey was carried out. Initially, we contacted a resident, and with their guidance, we visited households and individuals where data collection was possible. Only those participants who had experienced the 2024 flash flood in the study areas were selected. The level of flood impact was determined based on participants’ responses, such as whether they had been injured during the flood. As a result, we employed both purposive sampling techniques. We also selected this nonprobability sampling technique due to the remote areas participants. According to Morgan’s table, a sample size of 384 was deemed sufficient (37). One thousand nine hundred eighty-one participants were included in the final analysis.

We used Python (version 2.7; Beaverton, OR 97008, USA) and R (38, 39) for data management and statistical analysis. Descriptive statistics were calculated as needed. We employed multiple linear regression to examine the relationships between respondents’ demographic and flood-related loss and damage information with the three DASS sections. Variable selection was performed through simple linear regression analysis. Additionally, we assessed multicollinearity to refine our variable selection. We considered the three DASS sections as dependent variables, while sociodemographic factors and flood-related loss and damage information were treated as independent variables. As a result, we constructed four separate regression models (independent vs. dependent variables) in the multiple linear regression analysis. We also computed the three DASS labels based on the categories in Table 1.

### Ethical issue

This research was part of an approved study (Ref. No. KUECC-2022/06/16) by the Ethical Clearance Committee of Khulna University, Khulna, Bangladesh. The study adhered to all ethical guidelines outlined in the Declaration of Helsinki and its subsequent amendments (40). Informed consent was obtained from all participants and, where applicable, their legal guardians. For illiterate participants, the study’s purpose was explained to them verbally in a language they understood, and consent was obtained in accordance with the relevant ethical guidelines.

## Results and discussion

This section presents the results, followed by a discussion of the findings in relation to previous studies and their broader implications.

### Sociodemographic information

Table 2 presents the sociodemographic information. The gender distribution was nearly balanced, with 52.90% of participants being male and 47.10% female. Age-wise, the majority fell within the middle-aged categories, with 24.89% aged 36–45 years and 24.18% aged 46–55 years, while 14.99% were under 18. Most respondents (81.83%) were married, and educational attainment was generally low, with 54.97% not completing SSC and only 2.17% having higher education. Regarding occupation, 40.74% were unemployed after the flood, and 19.49% were employed in agro-fishery work. Income levels were significantly impacted, with 55.53% reporting no specific income post-flood, while only 2.07% earned above BDT 30,000 per month. Geographically, respondents were evenly distributed across the three upazilas: Sonagazi (32.81%), Chhagalnaiya (33.47%), and Fulgazi (33.72%). Housing types in the study area were categorized as kacha (constructed primarily with temporary or natural materials such as mud, bamboo, or thatch), semi-pucca (a combination of temporary and durable materials, such as brick walls with tin roofs), and pucca (houses made of durable materials such as brick and reinforced cement, considered permanent structures). Most respondents (98.69%) lived with family, and nearly half (49.02%) resided in kacha houses, making them highly vulnerable to flood damage. Additionally, 81.93% had vulnerable family members such as children, pregnant women, or older individuals. Chronic diseases were not widely reported, with 91.07% stating they had none, and only 3.53% reported living with disabilities. Social satisfaction levels were notably high, with 89.65% expressing satisfaction; however, 6.56% reported being the least satisfied with their post-flood circumstances. These findings highlight the socioeconomic vulnerabilities exacerbated by the disaster, particularly in terms of housing, employment, and income stability.

**Table 2:** Sociodemographic information.

Features	*n* (%)
1. Gender	
Male	1,048 (52.90)
Female	933 (47.10)
2. Age group (year)	
Less than 18	297 (14.99)
18–25	52 (2.62)
26–35	264 (13.33)
36–45	493 (24.89)
46–55	479 (24.18)
>55	396 (19.99)
3. Marital status	
Married	1,621 (81.83)
Unmarried	333 (16.81)
Others (Divorced, Separated, Widowed, etc.)	27 (1.36)
4. Education	
Illiterate	147 (7.42)
Non-SSC	1,089 (54.97)
SSC	582 (29.38)
HSC	120 (6.06)
>HSC	43 (2.17)
5. Occupation	
Agro-fishery workers	386 (19.49)
Business	190 (9.59)
Wage Labor	174 (8.78)
Student	313 (15.80)
Employee	111 (5.60)
Unemployed	807 (40.74)
6. Monthly income (BDT; 1 USD = approximately 120 BDT)	
No specific income after the flood	1,100 (55.53)
Less than 15,000	441 (22.26)
15,000–29,999	399 (20.14)
30,000-49,999	41 (2.07)
7. Upazila (Subdistrict)	
Sonagzi	650 (32.81)
Chhagalnaiya	663 (33.47)
Fulgazi	668 (33.72)
8. Living with family	
Yes	1955 (98.69)
No	26 (1.31)
9. Housing type	
Kacha	971 (49.02)
Pucca	422 (21.30)
Semi-pucca	588 (29.68)
10. Vulnerable family member (child, pregnant woman, older person, etc.)	
Yes	1,623 (81.93)
No	358 (18.07)
11. Chronic disease	
Maybe	119 (6.01)
No	1804 (91.07)
Yes	58 (2.93)
12. Disability	
No	1911 (96.47)
Yes	70 (3.53)
13. Social satisfaction	
Least satisfied	130 (6.56)
Satisfied	1776 (89.65)
Very satisfied	75 (3.79)

### Flood related information

The 2024 flash flood in Feni, Bangladesh, was part of a broader flooding crisis that affected several districts across the country. This disaster was triggered by heavy rainfall and upstream water flows from India, exacerbated by a low-pressure system over the Bay of Bengal (9, 41). The flood had a severe impact on communities in Feni and the surrounding areas.

Table 3 shows that a significant majority of respondents (86.42%) experienced a flood for the first time during this event, yet they perceived their locations as moderately safe against floods (87.88%). However, this perception may stem from limited exposure or a lack of understanding of flood risks, rather than actual safety measures in place.

**Table 3:** Flood-related information.

Items	*n* (%)
1. Previous flood experience before the 2024 flood	
No	1712 (86.42)
Yes	269 (13.58)
2. Current place’s safety rating against flood	
Moderately safe	1741 (87.88)
Unsafe	236 (11.91)
Safe	4 (0.20)
3. Did you get any kind of socioeconomic support during a flood?	
No	1,089 (54.97)
Yes	892 (45.03)
4. Did you receive an early warning regarding flood evacuation?	
No	1963 (99.09)
Yes	18 (0.91)
5. How would you rate the early warning mechanism for floods in your locality?	
Sufficient	2 (0.10)
Insufficient	140 (7.07)
No early warning dissemination mechanism at all	1839 (92.83)
6. What was the duration of the 2024 flood in your locality?	
2–3 days	5 (0.25)
4–6 days	217 (10.95)
7–10 days	1,675 (84.55)
11 days or more	84 (4.24)
7. Did you evacuate to the shelter during the flood?	
No	472 (23.83)
Yes	1,509 (76.17)
8. What is the reason behind not going to the shelter?	
No dedicated flood shelter	451 (95.55)
Insufficient flood shelter	7 (1.48)
Others	14 (2.97)

Despite these perceptions, socioeconomic support provided during the disaster was inadequate; over half of those affected received no assistance (54.97%). Moreover, the early warning system was highly ineffective: nearly all respondents reported not receiving evacuation warnings (99.09%), and most noted that there was no early warning mechanism in their locality (92.83%).

The duration of the flood lasted between 7 and 10 days for most respondents (84.55%), with many evacuating to shelters (76.17%). However, those who did not evacuate cited the absence of dedicated shelters as their primary reason for staying behind (95.55%). These findings underscore an urgent need for improved flood preparedness measures across multiple fronts.

Recent reports indicate that approximately 5 million people were affected by these floods across several districts, including Feni (41, 42). The situation led to significant displacement, with thousands seeking refuge in shelters. Additionally, infrastructure, including roads and agricultural fields, was severely damaged, substantially impacting the livelihoods of many.

To effectively mitigate future impacts from similar events in regions like Feni, it is essential to develop robust early warning systems that ensure timely alerts reach all at-risk populations. Comprehensive socioeconomic support mechanisms should also be established to address both immediate needs following a disaster and long-term recovery efforts (43). Furthermore, constructing accessible, dedicated shelters equipped with necessary amenities near high-risk areas would significantly enhance community resilience against recurrent natural disasters such as flash floods. Overall, addressing these gaps through improved preparedness will be crucial for reducing casualties and damage, while fostering sustainable recovery processes in vulnerable regions, such as the Feni district in Bangladesh. By prioritizing these improvements now, communities can better withstand future flooding events and rebuild more resiliently afterwards.

### Damage and loss

The 2024 flash flood in Feni, Bangladesh, had a profound impact on the local community, causing widespread damage and significant disruptions to livelihoods, infrastructure, and public services (8). The flood resulted in extensive residential damage, with 93.64% of respondents reporting harm to their homes (Table 4). The agricultural sector was also severely affected; 74.86% experienced crop damage, and 45.99% lost livestock. Infrastructure was also heavily impacted, with road damage reported by 33.72% of respondents and destruction of local infrastructure by 26.20%.

**Table 4:** Damage and loss due to the 2024 flash flood.

Items	*n* (%)
1. Damage and loss due to the 2024 flood in your locality?	
Livestock	911 (45.99)
Infrastructure	519 (26.20)
Road	668 (33.72)
Grazing field	32 (1.62)
Standing crop	1,483 (74.86)
Culture fisheries	476 (24.03)
Open fisheries	64 (3.23)
Homestead gardening	508 (25.64)
Residential unit	1855 (93.64)
Sanitation	765 (38.62)
Others	8 (0.40)
2. Has your household’s income been affected due to the 2024 flood?	
Yes	1946 (98.23)
No	35 (1.77)
3. Is there any long-term impact on your livelihood due to the 2024 flood in your locality?	
Yes	742 (37.46)
No	1,239 (62.54)
4. Have you lost any of your family members due to the 2024 flood?	
Yes	4 (0.20)
No	1977 (99.8)
5. What types of social and public service disruptions did you face during the 2024 flood?	
Healthcare services	273 (13.78)
Education services	960 (48.46)
Transportation services (e.g., blocked roads, disrupted public transport)	1922 (97.02)
Public utilities (e.g., water supply, electricity outages, sanitation issues)	1970 (99.44)
Communication services (e.g., disrupted phone/internet services)	1773 (89.50)
6. Is there any mental health issue due to the 2024 flood in your locality?	
Yes	1933 (97.58)
No	48 (2.42)
7. Had your house been inundated during the 2024 flood?	
No	2 (0.10)
Yes	1979 (99.90)
8. Did you have access to safe drinking water during the flood?	
No	1,685 (85.06)
Yes	296 (14.94)
9. Did you face any type of food scarcity to provide food for your family during the 2024 flood?	
No	424 (21.40)
Yes	1,557 (78.60)
10. Have you been injured due to the flood?	
Yes	26 (1.31)
No	1955 (98.69)
11. Have you got any diseases due to the flood?	
Yes	162 (8.18)
No	1819 (91.82)
12. Did any of your family members get injured during the 2024 flood?	
Yes	83 (4.19)
No	1898 (95.81)
13. Did any of your family members get disease during the 2024 flood?	
Yes	572 (28.87)
No	1,409 (71.13)

Economically, the flood resulted in substantial income loss for nearly all households (98.23%), with many anticipating long-term consequences for their livelihoods (37.46%). While fatalities were relatively low (0.20%), social services faced significant disruptions, as nearly all households experienced outages in essential utilities, including water supply, electricity, and sanitation (99.44%). Transportation was also severely disrupted for most residents (97.02%).

Mental health issues emerged as a major concern following the disaster (4); psychological distress was reported by an overwhelming majority of respondents (97.58%). Almost every household faced home inundation (99.90%), resulting in a widespread lack of access to safe drinking water (85.06%) and food scarcity, affecting approximately three-quarters of the households surveyed (78.60%). Despite relatively low injury rates among both respondents themselves and their family members, there were notable reports of flood-related illnesses affecting approximately one-tenth of the families surveyed, either directly or indirectly through family members experiencing symptoms of disease after the flood.

### Severity of depression, anxiety, and stress

Our study indicates that a significant portion of respondents are experiencing severe symptoms of depression (52.80%), anxiety (44.17%), and stress (46.90%) (Table 5). These findings align with previous research that highlights the psychological distress caused by floods (4, 44). Even though over one billion people worldwide are estimated to live with mental health conditions, mainly anxiety and depression, the rates of severe symptoms due to flash floods documented in this study notably exceed both the global and regional estimates found in recent mental health literature (45, 46). While global averages for severe depressive or anxiety symptoms generally range from 23–27% among the general population and rise to 30–40% among disaster-affected groups, the rates in Feni are markedly higher than in most comparative crises (27, 47). Feni’s severe mental health burden far exceeds national and international post-disaster estimates, reflecting the compounded stress of abrupt displacement, loss of homes, and breakdown of livelihoods.

**Table 5:** Depression, anxiety, and stress labels among respondents.

Severity label	Depression *n* (%)	Anxiety *n* (%)	Stress *n* (%)
Normal	36 (1.82)	149 (7.52)	131 (6.61)
Mild	40 (2.02)	44 (2.22)	205 (10.35)
Moderate	354 (17.87)	423 (21.35)	700 (35.34)
Severe	1,046 (52.80)	875 (44.17)	929 (46.90)
Extremely severe	505 (25.49)	490 (24.73)	16 (0.81)

### Associated factors

The study on the mental distress and health outcomes of individuals affected by the 2024 flash flood in Feni, Bangladesh, highlights several key factors influencing distress levels (Table 6). Regarding mental health outcomes, depression was strongly linked to education levels, with illiterate individuals (*β* = 1.78, 95% CI: 0.65, 2.90) and those with higher education (*β* = 2.45, 95% CI: 0.80, 4.10) experiencing more depression, potentially due to differing coping strategies and awareness of the crisis. For anxiety, lower education levels were associated with higher anxiety (*β* = 2.29, 95% CI: 1.32, 3.25), as were individuals with more than higher secondary education (*β* = 2.11, 95% CI: 0.73, 3.49). Stress was also influenced by education, with illiterate individuals (*β* = 1.33, 95% CI: 0.29, 2.37) and those with higher education (*β* = 1.77, 95% CI: 0.28, 3.25) reporting higher stress levels. Studies have shown that educational attainment can have a complex and sometimes non-linear relationship with psychological distress following disasters. For instance, lower educational attainment often corresponds with limited access to coping resources, lower health literacy, and reduced awareness of psychosocial support mechanisms, contributing to heightened vulnerability to depression and anxiety after disasters (12, 49, 50). Illiterate individuals may also depend more on informal supports and fatalistic interpretations of disaster impacts, which may not adequately moderate psychological distress. Conversely, individuals with higher education levels may experience greater depression due to heightened cognitive awareness of losses, future uncertainties, and socioeconomic disruptions.

**Table 6:** Associated factors with psychological distress, depression, anxiety, and stress.

Features	β# (95% CI)		
	Model I	Model II	Model III
	Depression	Anxiety	Stress
Education			
Illiterate	1.78 (0.65, 2.90)^*^	2.29 (1.32, 3.25)^*^	1.33 (0.29, 2.37)^*^
Non-SSC	−0.46 (−1.33, 0.42)	0.24 (−0.51, 0.99)	−0.93 (−1.75, −0.12)^*^
SSC	0.52 (−0.39, 1.42)	1.19 (0.41, 1.97)^*^	0.02 (−0.82, 0.85)
HSC	Reference	Reference	Reference
>HSC	2.45 (0.80, 4.10)^*^	2.11 (0.73, 3.49)^*^	1.77 (0.28, 3.25)^*^
Upazila (Subdistrict)			
Sonagzi		−4.01 (−4.47, −3.55)^*^	−1.15 (−1.64, −0.67)^*^
Chhagalnaiya		Reference	Reference
Fulgazi		−3.40 (−3.84, −3.13)^*^	−2.06 (−2.53, −1.59)^*^
Living with family			
Yes	4.97 (3.06, 6.88)^*^		3.18 (1.47, 4.90)^*^
No	Reference		Reference
Housing type			
Kacha	Reference		Reference
Pucca	−0.12 (−0.67, 0.44)		−0.00 (−0.51, 0.51)
Semi-pucca	−0.01 (−0.49, 0.47)		−0.19 (−0.63, 0.26)
Vulnerable family member			
Yes	0.58 (0.04, 1.12)^*^		1.12 (0.62 to 1.63)^*^
No	Reference		Reference
Chronic disease			
No	−1.32 (−2.20, −0.43)^*^		−0.74 (−1.55, 0.08)
Maybe	Reference		Reference
Yes	−3.80 (−5.30, −2.30)^*^		−1.11 (−2.50, 0.27)
Disability			
No		Reference	
Yes		1.28 (0.31, 2.25)	
Social satisfaction			
Least satisfied		Reference	Reference
Satisfied		−1.66 (−2.38, −0.94)^*^	−1.52 (−2.29, −0.75)^*^
Very satisfied		−1.54 (−2.72, −0.36)^*^	−3.28 (−4.55, −2.00)^*^
Household income affected by flood			
Yes	4.76 (2.85, 6.67)^*^	1.67 (0.01, 3.32)^*^	5.47 (3.70, 7.24) *
No	Reference	Reference	Reference
Long-term impact on livelihood due to 2024 flood			
Yes	1.02 (0.58, 1.46)^*^	0.20 (−0.17, 0.58)	0.18 (−0.22, 0.59)
No	Reference	Reference	Reference
Mental health issues due to 2024 flood			
Yes	8.58 (6.91, 10.25)^*^	8.65 (7.20, 10.09)^*^	9.92 (8.39, 11.46)^*^
No	Reference	Reference	Reference
Access to safe drinking water during flood			
No	Reference	Reference	Reference
Yes	−1.54 (−2.21, −0.88)^*^	−2.20 (−2.77, −1.63)^*^	−1.63 (−2.24, −1.01)^*^
Food scarcity during 2024 flood			
No	Reference	Reference	Reference
Yes	1.36 (0.79, 1.94)^*^	0.78 (0.29, 1.27)^*^	1.19 (0.66, 1.72)^*^
Injured during flood			
Yes	−3.30 (−5.11, −1.50)^*^		
No	Reference		
Diseases during flood			
Yes	2.78 (2.03, 3.54)^*^	1.83 (1.18, 2.48)^*^	
No	Reference	Reference	
Family member injured during 2024 flood			
Yes		−1.91 (−2.97, −1.49)^*^	−2.61 (−3.55, −1.67)^***^
No		Reference	Reference
Family member got disease during 2024 flood			
Yes	0.12 (−0.34, 0.58)^*^		0.44 (0.01, 0.86)^*^
No	Reference		Reference

*p < 0.05; **p < 0.01; ***p < 0.001; β^#^ = Beta (Coefficient). The beta coefficient indicates how much the outcome variable varies for every one-unit variation in the predictor variable (48). CI, Confidence Interval.

Family dynamics also contributed to depression levels, as living with family (*β* = 4.97, 95% CI: 3.06, 6.88) and having vulnerable family members (*β* = 0.58, 95% CI: 0.04, 1.12) were associated with higher depression, emphasizing the role of caregiving responsibilities in disaster-related distress. These findings highlight the intricate relationship between caregiving responsibilities and psychological well-being during disaster recovery. In post-flood environments where housing is damaged, income sources are disrupted, and access to healthcare is limited, caregivers often bear additional emotional and physical burdens (51). This strain can exacerbate stress and depressive symptoms, particularly among women and older adults who traditionally assume caregiving roles in Bangladeshi households. Moreover, cultural expectations surrounding familial duty and interdependence, while generally protective, can become stressors when resources are scarce, and the safety of dependents is threatened. Given that the 2024 floods disproportionately affected families living in low-lying and economically vulnerable regions, the psychological toll of caregiving likely compounded other risk factors such as economic loss, housing instability, and exposure to trauma. These findings highlight the need for psychosocial interventions that not only address individual mental health but also incorporate family-based support mechanisms, community caregiving networks, and targeted assistance for households with vulnerable dependents during disaster recovery efforts.

Chronic disease was again a protective factor, with individuals with chronic conditions reporting lower depression levels (*β* = −3.80, 95% CI: −5.30, −2.30), supporting the hypothesis that individuals with chronic conditions may possess enhanced resilience. Although previous research generally shows that individuals with chronic illnesses tend to experience greater psychological distress (52), our findings revealed the opposite pattern, participants with chronic conditions reported lower levels of depression. This counterintuitive result may reflect a form of psychological adaptation or resilience developed through long-term illness management. Individuals living with chronic conditions often acquire coping mechanisms, self-regulation skills, and established access to healthcare that can buffer emotional distress during acute crises. This may also represent a “response shift” phenomenon, in which individuals with chronic illnesses perceive disaster-related stressors as less severe relative to their ongoing health challenges. This interpretation underscores the importance of considering baseline health status when assessing mental health outcomes following disasters. While chronic illness typically increases vulnerability to physical hardship, in this case, it may also cultivate adaptive resilience that moderates depressive responses to acute environmental shocks.

Geography also played a role, with lower anxiety observed in residents of Sonagzi (*β* = −4.01, 95% CI: −4.47, −3.55) and Fulgazi (*β* = −3.40, 95% CI: −3.84, −3.13) compared to those from Chhagalnaiya. Similar results were also found in the case of stress, where residents of Sonagzi (*β* = −1.15, 95% CI: −1.64, −0.67) and Fulgazi (*β* = −2.06, 95% CI: −2.53, −1.59) experienced lower stress than those in Chhagalnaiya, further underlining the importance of community cohesion in mitigating stress. These upazilas, although all located within Feni District, experienced differing flood severity and recovery dynamics during the 2024 flash floods. However, within-district differences in elevation, infrastructure resilience, and community organization likely contributed to heterogeneous psychosocial outcomes. These findings underscore the significance of local geographic and infrastructural contexts in influencing mental health outcomes following floods. Even within the same district, variations in exposure, physical protection, and recovery support can produce markedly different psychological trajectories. They also underscore the potential of place-based resilience, where communities with stronger disaster preparedness mechanisms and social cohesion demonstrate lower post-disaster anxiety. Such geographically sensitive analyses are crucial for targeting mental health interventions and allocating resources effectively in disaster-affected regions of Bangladesh.

High levels of anxiety were found among individuals reporting widespread mental health issues in their locality (*β* = 8.65, 95% CI: 7.20, 10.09), reflecting the importance of community-level distress in exacerbating personal anxiety. When entire neighborhoods experience displacement, livelihood loss, or prolonged uncertainty, the cumulative emotional atmosphere can amplify anxiety through mechanisms of social contagion and shared trauma. In densely populated flood-affected areas of eastern Bangladesh, where the study reported extensive housing destruction and displacement (27, 53), social networks that typically provide emotional support may instead become channels for the transmission of fear and hopelessness. This finding reinforces the growing recognition that mental health in disaster settings is not solely an individual outcome, but a community-level phenomenon shaped by collective experiences and social cohesion.

Unexpectedly, reduced access to safe drinking water (*β* = −2.20, 95% CI: −2.77, −1.63) and having a family member injured during the flood (*β* = −1.91, 95% CI: −2.97, −1.49) were associated with lower anxiety levels. This counterintuitive pattern may reflect adaptive psychological mechanisms that arise when individuals face immediate, tangible hardships. During acute crises, attention often shifts from generalized worry to specific, goal-oriented coping behaviors, such as securing water, protecting family members, or accessing relief resources. This focused action can temporarily reduce anxiety by providing a sense of purpose and control amid uncertainty.

A comparison with previous studies reveals both similarities and key differences in the mental health outcomes experienced by flood survivors. Similar to other studies, who found significant psychological distress among survivors of the flash flood, our study also reports high rates of severe depression, anxiety, and stress among respondents (4, 27, 54–56). This aligns with previous research in other countries that identified a heightened vulnerability to mental health disorders following disasters (16, 57–62). The larger sample size in our study enhances the robustness of our findings and offers greater generalizability to similar flood-prone regions in Bangladesh.

Furthermore, our study extends the findings of previous research by identifying specific sociodemographic risk factors, such as education level, housing type, and social satisfaction, that significantly influence mental health outcomes post-flood. For instance, while other studies (4, 27, 54) primarily focused on livelihood impacts and gender as a predictor of distress, our study presents a more multidimensional framework, showing that factors like family dynamics, access to social support, and pre-existing health conditions contribute significantly to psychological distress. These findings suggest that future research should consider not only economic factors but also psychosocial dimensions in understanding disaster-induced mental health outcomes.

Building on our prior investigation (27), which utilized the Kessler Psychological Distress Scale (K-10) with a smaller sample size, this study represents a substantial methodological and conceptual extension. The earlier study primarily examined how livelihood disruption, displacement, and occupational loss contributed to emotional distress using the K-10. In contrast, the present study employs multiple regression models and a larger sample size with validated psychometric measures (DASS-21) to quantify specific dimensions of depression, anxiety, and stress, while identifying demographic and contextual risk factors. By integrating livelihood disruption with individual-level mental health outcomes, this study advances the literature by presenting a multidimensional framework that connects socioeconomic vulnerability, resilience, and psychological wellbeing in post-disaster contexts. Together, both studies offer a continuum of evidence, from livelihood impact to measurable mental health outcomes, that deepens understanding of disaster-induced psychological risk in Bangladesh.

Overall, the findings of this study underscore the multifaceted nature of mental distress following the 2024 flash flood in Feni, Bangladesh. Individual, familial, and community-level factors jointly shaped psychological outcomes, reflecting the complex interplay between vulnerability and resilience in post-disaster contexts. While educational, geographic, and social variables influenced depression, anxiety, and stress in distinct ways, the results also highlight that adaptive coping and resilience can emerge even amid profound disruption. These insights underscore the need for a comprehensive, context-sensitive mental health strategy in disaster management, one that integrates psychosocial support with community-based recovery, promotes inclusive mental health services for vulnerable populations, and enhances local preparedness systems. Embedding mental health interventions within disaster risk reduction and recovery programs can help ensure that affected populations are not only physically rehabilitated but also psychologically supported to rebuild their lives and resilience in the face of future climate-induced disasters in Bangladesh.

## Limitations and strengths

Despite its significant contributions, this study has certain limitations that should be acknowledged. First, the reliance on a cross-sectional design restricts the ability to establish causal relationships between flood exposure and mental health outcomes. A longitudinal study would provide a clearer understanding of how mental health conditions evolve following a disaster. Second, the use of nonprobability sampling may have introduced selection bias, as individuals more affected by the flood or those willing to participate may be overrepresented, limiting the generalizability of the findings. Additionally, the study relied on self-reported data for mental health assessments, which could be affected by recall bias and social desirability bias, potentially leading to over- or underestimation of psychological distress. Furthermore, while the study controlled for several sociodemographic and environmental factors, other confounding variables, such as pre-existing mental health conditions and access to mental health services before the disaster, were not comprehensively addressed. Finally, the research was geographically confined to the Feni District, meaning that its findings may not fully capture the experiences of flood-affected populations in other regions of Bangladesh or countries with different socio-environmental contexts.

Despite these limitations, the study presents several strengths that contribute to its robustness and relevance. The research provides valuable insights into the mental health impacts of flooding, particularly in a region where such effects have been underexplored. The use of validated psychological assessment tools, such as the DASS-21, enhances the reliability and comparability of the findings. Additionally, the large sample size increases the statistical power of the study and allows for a more comprehensive analysis of mental health outcomes across different sociodemographic groups. Furthermore, the study highlights critical factors influencing mental health, such as age, social support, and economic loss, which can inform targeted interventions and policy recommendations for disaster preparedness and response. By addressing an important research gap, this study serves as a foundation for future research on the mental health consequences of disasters in flood-prone regions.

## Recommendations

### Enhancing mental health support services

The findings of this study highlight the urgent need for accessible and community-based mental health support services for flood survivors. Establishing mental health programs within affected communities can provide counselling and psychological first aid to individuals experiencing post-traumatic stress, anxiety, and depression. Mental health services should be integrated into primary healthcare facilities to ensure easy access for vulnerable groups. Additionally, training healthcare professionals and community volunteers in mental health crisis intervention and trauma-informed care can improve the overall mental health response in disaster-prone areas.

### Strengthening disaster preparedness and response

A well-developed disaster preparedness and response system can significantly reduce the mental health impact of floods. Implementing an effective early warning system will allow communities to take necessary precautions before flooding occurs. Moreover, evacuation plans should be improved by ensuring that shelters are adequately equipped with clean water, food, and healthcare services. Investments in flood-resistant infrastructure, such as elevated housing, reinforced embankments, and improved drainage systems, will also help mitigate future flood-related damages. Strengthening these measures will ensure that communities are better prepared to handle the psychological and physical challenges posed by recurrent flooding.

### Addressing socioeconomic vulnerabilities

Floods often exacerbate existing socioeconomic inequalities, leaving many individuals without stable income or resources for recovery. To address these challenges, financial assistance programs should be implemented to support those who have lost their livelihoods. Additionally, vocational training initiatives can help displaced individuals develop new skills and re-enter the workforce. Strengthening social safety nets, such as conditional cash transfers and food assistance programs, will provide much-needed relief to the most vulnerable groups, including women, older adults, and individuals with disabilities. By addressing these socioeconomic vulnerabilities, affected communities can recover more effectively and build resilience against future disasters.

### Community awareness and capacity building

Raising awareness about mental health and disaster preparedness can empower communities to respond more effectively to floods. Public education campaigns should be conducted to inform individuals about coping mechanisms, stress management strategies, and available mental health resources. Community-based disaster preparedness drills can also improve residents’ ability to respond to emergencies and reduce panic during disasters. Furthermore, the establishment of local disaster response committees will enhance coordination among community members, ensuring a more organized and efficient disaster response. Strengthening community awareness and capacity building will help foster resilience and reduce the long-term psychological impact of floods.

### Policy and institutional interventions

Integrating mental health and psychosocial support into national disaster management policies is essential for a holistic approach to disaster response. Policymakers should prioritize the mental health of flood survivors by ensuring that psychological support is a key component of disaster relief programs. Additionally, regional cooperation with neighbouring countries is necessary to regulate water release from upstream dams, reducing the likelihood of sudden and severe flooding. Enforcing land-use policies to prevent construction in flood-prone areas will also help mitigate future risks. Strengthening institutional frameworks and policies will enhance the overall effectiveness of disaster management and mental health interventions.

### Further research and data collection

To develop more effective interventions, further research is needed to assess the long-term mental health impacts of flooding on different population groups. Conducting longitudinal studies will provide valuable insights into how psychological distress evolves and what factors contribute to recovery. Establishing a national database to track flood-related mental health issues can help inform targeted mental health and disaster response strategies. Additionally, interdisciplinary research exploring the intersection of climate change, disasters, and mental health can contribute to more comprehensive policy solutions. Continued research and data collection will ensure that mental health remains a priority in disaster resilience planning.

## Conclusion

The findings of this study underscore the significant mental health burden experienced by flood-affected individuals in Feni District following the 2024 flash flood. The high prevalence of psychological distress, including depression, anxiety, and stress, highlights the urgent need for targeted mental health interventions. Vulnerable groups such as women, older adults, and individuals with chronic illnesses were found to be at a higher risk of developing mental health disorders, emphasizing the importance of tailored support programs. Additionally, social satisfaction played a crucial role in mitigating distress, indicating that strengthening social networks and community-based mental health initiatives could be effective in reducing the psychological impact of floods. Beyond mental health, the study also revealed critical gaps in disaster preparedness and response. The absence of an effective early warning system, inadequate evacuation shelters, and limited access to socioeconomic support exacerbated the challenges faced by affected individuals. Addressing these issues through improved disaster management policies, infrastructure investments, and community awareness programs is essential to enhancing resilience in flood-prone areas. Moreover, integrating mental health care into disaster response frameworks will ensure that psychological wellbeing is prioritized alongside physical recovery efforts. Given the increasing frequency and severity of climate-related disasters, there is an urgent need for a comprehensive and multi-sectoral approach to flood management. Strengthening policy interventions, enhancing social safety nets, and expanding research on disaster-related mental health impacts are necessary to build long-term resilience. Future studies should focus on the long-term mental health consequences of floods and assess the effectiveness of intervention strategies. By addressing both the immediate and long-term challenges posed by floods, stakeholders can work toward a more sustainable and resilient disaster management system in Bangladesh. This study contributes valuable insights into the mental health impacts of flooding and provides evidence-based recommendations for policymakers, health professionals, and disaster management authorities. Ensuring proactive measures, community involvement, and mental health integration in disaster response plans will be critical in mitigating the psychological impact of future disasters.

## Data Availability

The raw data supporting the conclusions of this article will be made available by the authors, without undue reservation.
