# Correction: Mental health consequences of the 2024 Feni flash flood in Bangladesh: prevalence and risk factors

**DOI:** 10.3389/fpubh.2026.1787889

**Published:** 2026-01-27

**Authors:** Md Mostafizur Rahman, Samantha Alam, Ifta Alam Shobuj, Md. Mehedi Hasan Santo, Md. Tanvir Hossain, Farzana Rahman, Md. Kaium Hossain, Edris Alam, Md. Kamrul Islam

**Affiliations:** 1 Department of Disaster Management and Resilience, Faculty of Arts and Social Sciences, Bangladesh University of Professionals, Dhaka, Bangladesh; 2 Sociology Discipline, Social Science School, Khulna University, Khulna, Bangladesh; 3 Department of Computer Science and Engineering, Independent University, Dhaka, Bangladesh; 4 School of Business and Economics, United International University, Dhaka, Bangladesh; 5 Department of Geography and Environmental Studies, University of Chittagong, Chittagong, Bangladesh; 6 Faculty of Resilience, Rabdan Academy, Abu Dhabi, United Arab Emirates; 7 Department of Civil and Environmental Engineering, College of Engineering, King Faisal University, AlAhsa, Saudi Arabia

**Keywords:** mental health, flash flood, psychological distress, disaster resilience, Bangladesh, disaster preparedness

The following funding information in the Funding statement was erroneously omitted: “Funding for the article processing charge (APC) was partially covered by the Deanship of Scientific Research, Vice Presidency for Graduate Studies and Scientific Research, King Faisal University, Saudi Arabia (Grant: KFU A372).”

The original version of this article has been updated.

